# A plausible link between the time-on-task effect and the sequential task effect

**DOI:** 10.3389/fpsyg.2022.998393

**Published:** 2022-10-26

**Authors:** Thomas Mangin, Michel Audiffren, Alison Lorcery, Francesco Mirabelli, Abdelrhani Benraiss, Nathalie André

**Affiliations:** ^1^Centre de Recherches sur la Cognition et l’Apprentissage (CeRCA), UMR CNRS 7295, Université de Poitiers, Poitiers, France; ^2^Faculté de Médecine, École de Kinésiologie et des Sciences de l’Activité Physique (EKSAP), Université de Montréal, Montreal, QC, Canada; ^3^Centre de Recherche de l’Institut Universitaire de Gériatrie de Montréal (CRIUGM), Montréal, QC, Canada

**Keywords:** boredom, ego depletion, mental fatigue, sequential task protocol, time-on-task

## Abstract

Mental fatigue can be studied by using either the time-on-task protocol or the sequential task protocol. In the time-on-task protocol, participants perform a long and effortful task and a decrease in performance in this task is generally observed over time. In the sequential task protocol, a first effortful or control task is followed by a second effortful task. The performance in the second task is generally worse after the effortful task than after the control task. The principal aim of the present experiment is to examine the relationship between these two decrements in performance while concomitantly using a sequential task protocol and assessing the performance of the first effortful task as a function of time-on-task. We expect a positive correlation between these two decrements in performance. A total of 83 participants performed a 30-min fatiguing mental task (i.e., a modified Stroop task) or a control task followed by a time-to-exhaustion handgrip task. As expected, this protocol combining the time-on-task and sequential task protocols allowed us to observe (1) a decrease in performance over time during the Stroop task, (2) a worst performance in the handgrip task after the Stroop task by comparison to the control task, (3) a positive correlation between these two effects. The decrease in performance during the Stroop task also correlated with the subjective measures of boredom and fatigue, whereas the detrimental effect observed in the handgrip task did not. Our findings suggest that the two fatigue-related phenomena share a common mechanism but are not completely equivalent.

## Introduction

Mental fatigue can be defined as a psychological state, whose symptoms are a feeling of tiredness and a lack of energy. These symptoms are generally associated with a reduction in performance and/or an increase in mental effort engaged in the ongoing task and experienced during or after prolonged periods of effortful cognitive activity (Ackerman, 2011; Van Cutsem et al., 2022). This phenomenon impacts society as a whole by reducing performances in many different domains, such as decision-making (Osgood, 2019), planification (Sjåstad and Baumeister, 2018), alcohol abuse (Muraven et al., 2002), or different physical activities (Pageaux and Lepers, 2018).

Two protocols have been developed by experimental psychologists to induce and study mental fatigue. In the late 1960s, researchers used the time-on-task protocol to study the decrease in vigilance when the task lasted for a long time (Mackworth, 1968). Later, this protocol was used to measure the performance decrease in other cognitive tasks, such as reaction time tasks (Boksem et al., 2005; Simon et al., 2020; Arnau et al., 2021; Matuz et al., 2021). Generally, reaction time, misses and false alarms increase with time-on-task. For example, after 3 h of the experimental task, reaction time increased by 5.2%, and misses increased by 71% (Boksem et al., 2005). The lengthening of reaction time and/or the increase in the number of decision errors with time-on-task is attributed to mental fatigue. In this protocol, the fatiguing task generally lasted more than 30 min. Different laboratory tasks aiming to induce mental fatigue have been used: The Psychomotor Vigilance task, AX-Continuous performance test, Stroop task (Smith et al., 2019), Simon task (Arnau et al., 2021), or Go/No-Go Task (Melo et al., 2017). More ecological tasks have also been used with the time-on-task protocol, such as driving tasks in simulator (e.g., Van Der Hulst et al., 2001; Marando et al., 2022) or typewriting (de Jong et al., 2020).

Later in the 1990s, researchers in social psychology created and used the sequential task protocol to study the decrease in performance in a task that followed an initial depleting mental task (Baumeister et al., 1998; Muraven et al., 1998). This effect, often called ego depletion effect (Hagger et al., 2016; Blázquez et al., 2017; Dang et al., 2020), is generally obtained when participants perform a second effortful task after a first effortful task. More precisely, in one group or condition participants must perform a first effortful task (i.e., the depleting task), while in another group or condition they must carry out a control task requiring little mental effort. Just after the end of the first task, participants must perform a second effortful task that is similar in both groups or conditions (i.e., the dependent task). The decrease in performance observed in the dependent task when comparing the depleting group or condition with the control group or condition is interpreted as an indicator of ego or self-control depletion in the framework of the strength model of self-control (Baumeister et al., 2007; Hagger et al., 2010). The sequential task protocol has been also used in the field of sport sciences to study the effect of mental fatigue induced by a first cognitive task on a subsequent physical task (see recent reviews on this topic: Van Cutsem et al., 2017b; Giboin and Wolff, 2019; Brown et al., 2020; Hunte et al., 2021). In order to dissociate the decrease of performance observed after the first effortful task from a specific theory or area of research, a more neutral term than “ego depletion” was used throughout the manuscript. In that perspective, the term “sequential task effect” was preferred and used thereafter. This sequential task effect has been observed in cognitive tasks, i.e., reaction time tasks (Muraven and Slessareva, 2003; Moller et al., 2006; Dang et al., 2020), or physical tasks, i.e., resistance or endurance exercise until exhaustion (Pageaux et al., 2013; Boat and Taylor, 2017; Brown and Bray, 2017).

The duration of the depleting task in the sequential task protocol can be short (3–10 min) or long (>30 min) and the sequential task effect can be observed with both durations (see Giboin and Wolff, 2019). By contrast, the duration of the fatiguing task in the time-on-task protocol is generally long (>30 min) and the time-on-task effect requires a long fatiguing task to be observed. We assume that when the duration of the depleting task in the sequential task protocol and the duration of the fatiguing task in the time-on-task protocol are long, then the sequential task effect and time-on-task effect share commonalities related to mental fatigue. However, if the duration of the depleting task is short, other mechanisms, not related to mental fatigue can induce the sequential task effect, such as a shift in motivation (Inzlicht et al., 2014), or beliefs on human mental resources (Job et al., 2010). These two latter mechanisms can also contribute to negative effects observed with long depleting tasks, but according to the integrative model of effortful control (André et al., 2019), it is assumed that mental fatigue increases over time when effortful control is required.

Then, it is possible to examine the performance during a long depleting task as a function of time-on-task within a sequential task protocol. In such a protocol, the time-on-task effect can be observed in addition to the sequential task effect, which is obtained by comparing the performance in the dependent task as a function of condition (i.e., depleting vs. control condition). More than 50 studies already used a protocol allowing to examine the two effects of interest in the same experiment (see Supplementary Table A1). With such a protocol, it can be assumed that if the time-on-task effect and sequential task effect share a similar causal mechanism (e.g., a weakening of the capacity to exert effortful control), a correlation could be observed between the two effects: participants exhibiting a larger time-on-task effect show a larger sequential task effect. Showing such a correlation between these two well-documented phenomena would be the first step demonstrating that they share commonalities. Among the 56 studies that used a sequential task protocol with a long depleting task, only 10 reported a time-on-task effect on the performance of the depleting task (see the systematic review on this point reported in the Supplementary Section 8). Among these 10 studies, 8 observed a time-on-task effect and a sequential task effect in the same experiment, but none of them examined the correlation between these two effects (Marcora et al., 2009; Smith et al., 2015; Veness et al., 2017; Brown and Bray, 2019; Van Cutsem et al., 2019; Hachard et al., 2020; Morris and Christie, 2020; Weerakkody et al., 2021), probably because of a small sample size. The principal aim of the present experiment is then to highlight a positive correlation between the time-on-task effect and the sequential task effect but with a larger sample size than previous studies.

Nevertheless, the use of a long and monotonous depleting task introduces other confounding factors, such as boredom that can induce a decrease of performance over time in the same way than mental fatigue (e.g., Pattyn et al., 2008). Boredom, which can be defined as “an aversive state of wanting, but being unable, to engage in satisfying activity” (Eastwood et al., 2012, p. 482), can be associated with effortful control and a high opportunity cost when the participant remains engaged on the task in spite of his/her desire to stop the task (Kurzban et al., 2013). For instance, in the sustained attention literature, boredom is correlated with attention failures that increase with time-on-task (Raffaelli et al., 2018). In the ego-depletion literature, boredom contributes to variance in the performance decline observed in the dependent task after the depleting task (Wolff and Martarelli, 2020). Consequently, boredom appears to be an important variable that needs to be controlled in both protocols previously cited. Moreover, boredom could mediate the relationship between the time-on-task effect and sequential task effect. For instance, boredom felt during the depleting task may lead to a decrease in performance with time-on-task in the depleting task and then a decrease of motivation to perform the dependent task. For instance, in a previous experiment (Mangin et al., 2021), boredom felt during the depleting task negatively correlated with the motivation to perform the second task (*r* = −0.364, *p* = 0.009). An additional aim of the present study is to examine this possible mediating role of boredom in the relationship between the time-on-task effect and sequential task effect.

Based on what has been said above, our main hypothesis assumes that there is a positive correlation between the time-on-task effect observed in the depleting task and the sequential task effect observed in the subsequent dependent task: the higher the decrement in performance over time during the depleting task is, the larger the sequential task effect when comparing the performance of the dependent task after the depleting task and after the control task. Our secondary hypothesis claims that boredom felt during the depleting task will correlate with the time-on-task effect and sequential task effect and will explain, at least in part, the correlation between these two effects (i.e., boredom is a variable that mediates these two effects).

## Method

### Participants

A total of 83 participants took part in this experiment (57 females, mean age = 26.30 years, *SD* = 12.07) in exchange for course credits for the students of the University of Poitiers and 30 € for non-student participants. The method used to determine the sample size is detailed in the Supplementary Section 1. The participants received an oral explanation about the protocol and then signed an informed consent form. The experiment was approved by the local ethics committee (n°CER-TP 2019-11-08). We used 2.5 absolute deviations around the median to consider data as outliers (Leys et al., 2013). Four participants were excluded because their performances deviated from the median by more than 2.5 absolute deviations.

### Procedure

**FIGURE 1:**
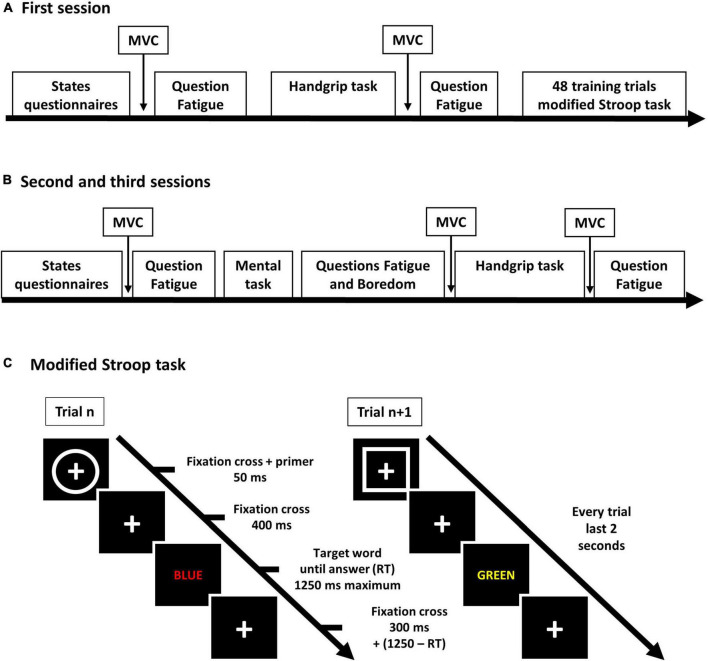
Design of the experiment. **(A)** Time course of the first session. **(B)** Time course of the second and third sessions. **(C)** Time course of the trials during the modified Stroop task. MVC, maximal voluntary contraction measured with the handgrip.

In this experiment, participants experienced three distinct sessions. The participants were instructed to avoid alcohol or any drugs 24 h before the experiment and caffeine 6 h before. Participants were also instructed to avoid strenuous activity 12 h before experimental sessions. During the first session, participants performed a handgrip task until exhaustion and then were familiarized with a modified Stroop task (Figure 1A). During the second and the third sessions (Figure 1B), participants first performed a mental task that was either a modified Stroop task or watching a documentary (i.e., “Earth” by Fothergill and Linfield, 2007). The order of these sessions was randomized. This first task was followed by the handgrip task until exhaustion. In these sessions, immediately after the mental task, participants had to indicate the boredom they felt during the task. Furthermore, they had to indicate their feeling of fatigue before and after each task. Fatigue and boredom were evaluated using a visual analog scale (Wewers and Lowe, 1990), ranging from 0 to 100%. Participants were blind to the objectives and hypotheses of the experiment. At the end of the last session, participants were debriefed about the experiment. First, they could ask as many questions as they would. Then the hypotheses and the name of the documentary were given to them. We also asked the participants not to discuss about the experiment outside the laboratory. If they had any further questions, they were told to send an email to the experimenter. The non-students were paid by the laboratory secretariat office.

The modified Stroop task was computerized, composed of 888 trials and lasted 29.6 min. The time course of a trial was as follows (Figure 1C): first, a fixation cross appeared on the screen enclosed by a square or a circle for 50 ms. It was followed by a fixation cross for 400 ms. Then, a color word appeared in the middle of the screen and lasted until the participant answered or, in case of omission, for a maximum time limit of 1,250 ms. After the word disappeared, a fixation cross was displayed in the middle of the screen and remained at this location until a time limit of 2,000 ms was reached for each trial. The words were color names in French (i.e., Red, Blue, Green, and Yellow), with an unmatching color ink (e.g., Red written in blue). When the square was displayed (50% of the trials, randomized), participants were instructed to read the word. When it was the circle, they had to name the color of the ink. Participants gave their answer orally because this modality generated more interference, and it was more difficult to inhibit reading than the manual answering version of the task (MacLeod, 1991). A first microphone connected to Eprime allowed to detect the beginning of the vocal responses of the participants for the calculation of the reaction times. A second microphone recorded the vocal response allowing to determine the accuracy of the response. For the control mental task, participants watched the first 30 min of the documentary “Earth.”

The handgrip task until exhaustion was performed using a hand dynamometer (TSD121C, BIOPAC), and the force signal was recorded using the MP160WSW unit and AcqKnowledge software (BIOPAC Systems Inc., Goleta, USA). Participants were instructed to maintain a submaximal isometric contraction (13% of maximal voluntary contraction, MVC) until exhaustion. To perform this task, a circle was displayed on a screen in front of them, and 360° represented their MVC measured at the beginning of the first session. Feedback about the force produced by the participant was provided by a gauge needle. Participants had to maintain the needle in a target portion of the gauge that represented 12–14% of their MVC. The task stopped when participants released the handgrip or when they stayed under the 12% limit (i.e., under the target zone) for more than 2 s. The MVC was measured at the beginning of each session by demanding to the participant to squeeze the handgrip as strong as they could during 3 s. This measure was repeated with a rest interval of 30 s until their force did not increase from the previous trials. A more detailed description of this task is provided in Mangin et al. (2021).

### Measure of performance and composite scores

To measure the performance in the modified Stroop task, we used the inverse efficiency score (IES; Akhtar and Enns, 1989; Bruyer and Brysbaert, 2011; Liesefeld and Janczyk, 2019), an index of performance that combines speed and accuracy. This score is calculated as follows: IES = mean reaction time for correct responses/(1 – proportion of decision errors). This calculation combines the reaction time and the error rate of the participants, two of the most important indicators of performance. The IES was calculated while averaging the performances of both types of trial (i.e., “naming the color of the ink” vs. “reading the color word”). We also divided the 30 min of the modified Stroop task into four parts of 7.5 min and calculated the IES score for each part.

We also created four composite scores. First, for the effect of time-on-task, we subtracted the IES score of the fourth part from the first part, and then divided this difference by the IES score of the first part, for each participant. This composite score represented the ratio of change in performance for each participant, depending on their performance at the beginning of the task. A negative ratio means that there was a decrease in performance from the beginning to the end of the Stroop task. Second, for the sequential task effect, we subtracted the performance in the handgrip task until exhaustion after the control task from the performance after the Stroop task, and then divided this difference by the performance after the control task for each participant. This composite score represented the ratio of change in performance in the handgrip task until exhaustion. A negative ratio means that there was a decrease in performance in the handgrip task after the Stroop task compared to after the control task. Third, we subtracted the boredom felt during the control task from the boredom felt during the Stroop task. Finally, we subtracted the fatigue felt after the control task from the fatigue felt after the Stroop task.

### Statistical analyses

We performed the statistical analyses using Jasp 0.16.2.0. The statistical significance was set at alpha level = 0.05. The correlations are Pearson’s r and the *t*-tests are Student’s *t*-test. We tested the sphericity with the Mauchly test when a repeated-measure ANOVA included a within-subjects factor with more than two levels. If the sphericity was violated, then we applied the Huynh-Feldt adjustments. All *post-hoc* comparisons were corrected following the Bonferroni procedure.

## Results

### Performance in the modified Stroop task

**FIGURE 2:**
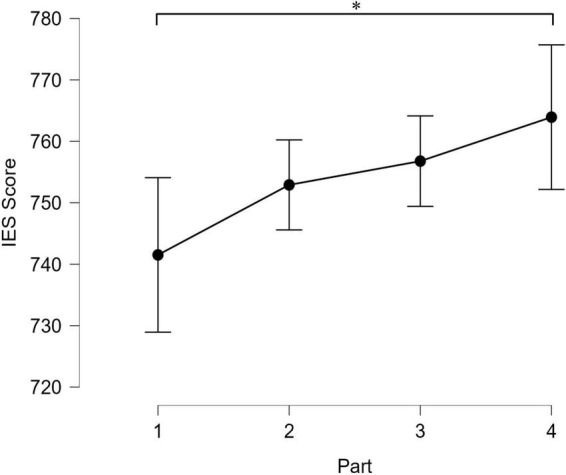
The inverse efficiency score in milliseconds as a function of time-on-task. Part 1 = the first 7.5 min, Part 2 = from 7.5 to 15 min, Part 3 = from 15 to 22.5 min, Part 4 = from 22.5 to 30 min. Error bars represent the 95% confidence interval. *Significant difference between the conditions.

An ANOVA with time-on-task (Part 1, Part 2, Part 3, Part 4) as a within-subjects factor was conducted on the IES score. This effect of time-on-task reached significance: *F*(2.04, 159.15) = 3.44, *p* = 0.034, η*^2^* = 0.042. The *post-hoc* comparison with Bonferroni correction indicated that the IES score of the first part (*M* = 741.5, *SD* = 114.9) was significantly lower than the IES score of the fourth part (*M* = 763.9, *SD* = 109.8): *t*(234) = 3.14, *p* = 0.011, *dz* = 0.20, IC_95_ [0.02, 0.38]. All other comparisons were not significant. However, a linear contrast confirmed that the performance decreased with time-on-task throughout all parts: *t*(234) = 3.15, *p* = 0.002, β = 15.91, IC_95_ [5.96, 25.86] (Figure 2). The effect of time-on-task on speed (mean reaction time) and accuracy (error rate) were presented separately in the Supplementary Sections 2–5 and two analyses were conducted: (1) while mixing the performance of the two types of trials (i.e., “naming the ink color” vs. “reading the color word”) and (2) while differentiating these two types of trials. These complementary analyses confirmed the results presented above.

To measure the sequential task effect, we conducted a paired sample *t*-test with the session (control vs. Stroop) as repeated measures on the performances in the handgrip task until exhaustion. Participants squeezed the handgrip longer in the control session (*M* = 7.26, *SD* = 3.74) than during the Stroop session (*M* = 6.66, *SD* = 3.50): *t*(78) = 3.23, *p* = 0.002, *d*_*z*_ = 0.36, IC_95_ [0.13, 0.59].

We also measured the boredom felt by the participants during the mental tasks (Stroop and video tasks). We found that the participants felt more bored during the modified Stroop task (*M* = 47.4, *SD* = 30.3) than during the video task (*M* = 21.8, *SD* = 20.0): *t*(78) = 7.32, *p* < 0.001, *d*_*z*_ = 0.82, IC_95_ [0.57, 1.08].

Concerning subjective fatigue, participants also felt more tired after the modified Stroop task (*M* = 44.7, *SD* = 26.7) than after the video task (*M* = 29.8, *SD* = 20.8): *t*(78) = 5.28, *p* < 0.001, *d*_*z*_ = 0.59, IC_95_ [0.35, 0.83].

**FIGURE 3:**
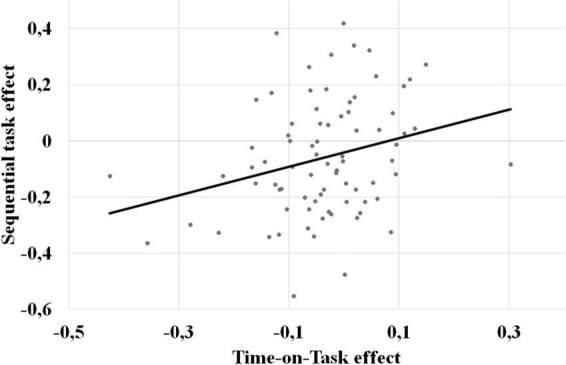
Correlation between the sequential task effect (*y*-axis) and the time-on-task effect (*x*-axis). A negative score on both axes means a decrease in performance.

The correlations between the four composite scores presented in the method section are reported in Table 1. Our results showed positive correlations between the time-on-task effect and sequential task effect (see Figure 3), and between the index of boredom and the index of fatigue. Negative correlations were also observed between the time-on-task effect and the boredom index and between the time-on-task effect and the fatigue index. We did not observe a correlation between the sequential task effect and the indices of boredom or fatigue. Furthermore, when we controlled for the boredom index in the correlation between the time-on-task effect and sequential task effect, the correlation became marginal: *r* = 0.217, *p* = 0.056. A complete mediation analysis is presented in the Supplementary Section 7.

**TABLE 1:** Correlation matrix of composite scores of sequential task effect, time-on-task effect, boredom, and subjective fatigue.

Variable	Sequential task	Time-on-task	Boredom
Time-on-task	*r* = 0.272, *p* = 0.015*		
Boredom	*r* = −0.216, *p* = 0.055	*r* = −0.332, *p* = 0.003*	
Fatigue	*r* = −0.067, *p* = 0.557	*r* = −0.231, *p* = 0.040*	*r* = 0.309, *p* = 0.006*

Sequential task effect = (performance in the handgrip task until exhaustion after the Stroop task minus performance after the control task) divided by performance after the control task. Time-on-task effect = (inverse efficiency score of the first part minus the inverse efficiency score of the fourth part) divided by the inverse efficiency score of the first part. Boredom = subjective measure of boredom after the Stroop task minus the subjective measure of boredom after the control task. Fatigue = subjective measure of fatigue after the Stroop task minus subjective measure of fatigue after the control task.

*Significant correlation between the variables.

## Discussion

In this experiment, we expected to observe a positive correlation between two well-known phenomena related to mental fatigue: the time-on-task effect and the sequential task effect. However, before examining the correlation between these two effects, it was necessary to observe these two effects. First, we successfully replicated the time-on-task effect, such that the performance in the depleting task worsened over time as it can be found in the literature (Esterman and Rothlein, 2019). The performance from the first part to the last part decreased by 3.7% on the inverse efficiency score. Second, we also successfully replicated the sequential task effect. Participants performed worse (i.e., 6.1% less) in the handgrip task until exhaustion after the modified Stroop task than after the video task. Third, we found that the modified Stroop task was more boring for the participants than the video task. Fourth, in the same way, participants reported more fatigue after the modified Stroop task. These last two results are consistent with the literature on the sequential task effect (Brown and Bray, 2017; Van Cutsem et al., 2017a; Wolff and Martarelli, 2020).

Then, as expected, we observed a weak but significant correlation between the time-on-task effect and sequential task effect. To our knowledge, this is the first time that a correlation between these two effects has been investigated and found. This correlation indicates that the more the performance declined throughout the depleting task, the larger the sequential task effect was (see Figure 3). This statistical relationship between the time-on-task effect and the sequential task effect can be interpreted in three different ways: (1) it suggests that these two performance declines are underpinned, at least in part, by a common mechanism (i.e., decline of performance in the depleting task has the same cause than the decline in performance observed in the dependent task); (2) it is fortuitous and due to pure chance; (3) it reflects the effect of a confounding factor (e.g., the sample of participants is composed of two sub-groups whose one individual characteristic is associated with one of the two variables involved in the correlation, for instance one of the sub-groups include more depressed participants and these participants are more sensitive to fatigue). However, our experiment did not allow us to examine which mechanism explains these two fatigue-related phenomena and to decide between these three explanations. The present experiment just pointed out on a possible link between two well-known effects in psychology that needs to be explained by neuroimaging studies.

As mentioned in the introduction, numerous studies used a sequential task protocol with a long depleting task to study the effect of mental fatigue induced by a first effortful task on a subsequent cognitive or physical task (see the systematic review in the Supplementary material). However, few of these studies have reported or observed the time-on-task effect and were able to calculate the correlation between the time-on-task effect and the sequential task effect. The lack of investigation of this correlation could be explained by three main reasons: (1) a small sample size in the vast majority of these studies making it difficult to examine the relationship between the two phenomena of interest given the large inter-individual variability in sensitivity to mental fatigue (89.29% of the studies with *N* < 30); (2) a depleting task that was not sufficiently practiced by the participants before the experiment and showed a practice effect or masked a decrement of performance as a function of time on task (8.93% of the studies observed a practice effect); (3) a depleting task that was not conceived to examine precisely the evolution of performance as a function of time on task (e.g., using social media on a smartphone, sport-based video-game; 8.93% of the studies).

In addition, boredom has been identified as an important factor to control in order to observe the sequential task effect (Wolff and Martarelli, 2020). In this study, we observed a non-significant but marginal negative correlation between boredom felt during the depleting or the control task and the sequential task effect. It is possible that this correlation can reach significance when increasing the study power. However, a negative correlation between the boredom index and the time-on-task effect was found. The higher the index of boredom was (i.e., higher boredom during the depleting task by contrast to the control task), the higher the decrease in performance in the Stroop task was (i.e., larger time-on-task effect). The boredom felt by the participants in the Stroop task but not in the control task can be due either to an overstimulation of participants perceiving the Stroop task as too difficult and/or a monotonous Stroop task (Raffaelli et al., 2018). Furthermore, boredom can cause difficulty concentrating on the ongoing task and lead to a non-optimal arousal level (Eastwood et al., 2012) that can be linked to attention failure (Raffaelli et al., 2018). In this way, participants who felt boredom during the Stroop task may have progressively disengaged effort from this task when they performed it. This progressive disengagement of effort can explain the decrease in performance throughout the Stroop task. As demonstrated in the results section, when controlling for the boredom index (i.e., the difference in feeling of boredom in the Stroop task and the control task) the correlation between the sequential task effect and time-on-task effect became marginal, meaning that boredom is partially involved in the common mechanism underpinning these two phenomena.

We also found a positive correlation between the boredom index and the fatigue index: the higher the boredom index was, the higher the fatigue index. This result is in line with the results of Milyavskaya et al. (2019), who found higher fatigue after 20 min of the boring task compared to the non-boring task.

Our results suggest that boredom contributes notably to the variance in the time-on-task effect. Boredom is linked with more activation of the default mode network (Danckert and Merrifield, 2018), which can explain the negative correlation with the time-on-task effect (i.e., the higher the boredom is, the larger the number of activations of the default mode network and the number of attentional lapses). The more frequent activation of the default mode network (DMN) during a task may be explained by a weakening of the ability of the salience network to keep the DMN disabled while maintaining activation of the executive control network (André et al., 2019).

### Limits and perspectives

In the current experiment, we showed that the time-on-task effect and sequential task effect shared a common percentage of variance. However, we do not know which mechanism was involved in the decrease in performance in both tasks. We know that boredom explains in part this decrease, supporting the hypothesis of a decrease in motivation (Bench and Lench, 2013; Tze et al., 2014; Mangin et al., 2021), but the correlation between the two effects remained marginal suggesting that another mechanism contributed to this correlation. If the same mental fatigue mechanism is implied in both tasks, then it should be possible to show its contribution in the two phenomena through a mediation analysis and observe it with psychophysiological or neuroimaging measures. For instance, a disengagement of effort during the depleting and dependent tasks could be examined by measuring the variations in sympathetic and parasympathetic activities through cardiac reactivity indices (Richter et al., 2016; Van Cutsem et al., 2022) or the variations in density of prefrontal theta waves (Klimesch, 1999; André et al., 2019).

The term “ego-depletion” is generally associated with a mechanism leading to the occurrence of this effect: the depletion of an internal resource, such as brain glucose (Gailliot and Baumeister, 2007). However, this mechanism is not plausible and has been criticized (e.g., Beedie and Lane, 2012; Kurzban et al., 2013; Lange and Eggert, 2014; Vadillo et al., 2016). The mechanism that receives the most attention from our team is the hypothesis of an action of brain adenosine released in situations of intense and persistent neuronal activity (Pageaux et al., 2014; Martin et al., 2018; André et al., 2019). This brain metabolite could decrease the efficiency of prefrontal pyramidal neurons and inhibit striatal dopamine, simultaneously reducing the capacity of prefrontal processing units to maintain effortful control and the motivation to exert effortful control (André et al., 2019). Positron emission tomography (PET) could allow us to test this hypothetical mechanism. Studies using such biomarkers in addition to behavioral and subjective measurements will be useful in the future to investigate the mechanisms underpinning mental fatigue.

Finally, in this article, we observed that the correlation between the boredom index and sequential task effect was marginal, although it has been predicted in another article that boredom plays a role in the sequential task effect by increasing the effort cost to perform the depleting task (Wolff and Martarelli, 2020). From this perspective, another study with a different depleting task (e.g., longer and more boring) should further examine the relationship between boredom felt during the depleting and control tasks and the size of the sequential task effect.

## Conclusion

The current experiment highlights that the participants with the higher deterioration in their performance during the fatiguing task (i.e., time-on-task effect) were also the participants that displayed the higher deterioration in the dependent task after the fatiguing task by comparison to after the control task (i.e., ego-depletion or sequential task effect). This result suggests that it is crucial to control the evolution of performance as a function of time-on-task in the depleting task because it can contribute to the subsequent sequential task effect. A plausible explanation of the link between these two effects, when the depleting task is long, is that they share a common underlying mechanism related to mental fatigue, but other explanations cannot be rejected. Finally, only a weak percentage of variance explains the link between the time-on-task effect and sequential task effect. Future experiments are required to find experimental conditions increasing the commonalities between these phenomena. For instance, it would be interesting to increase the cognitive control required by the depleting task (e.g., dual N-back task), to reduce as much as possible practice effects in the performance of the depleting task (e.g., participants must reach a performance plateau before starting the experiment) and to include a population of participants particularly sensitive to mental fatigue effects (e.g., participants with a low level of mental toughness). Such a protocol could allow to demonstrate that the two well-known phenomena attributed to mental fatigue are closely related.

## Data availability statement

The raw data supporting the conclusions of this article will be made available by the authors, without undue reservation.

## Ethics statement

The studies involving human participants were reviewed and approved by the Comité d’Éthique pour les Recherches Impliquant la Personne Humaine – Universités de Tours et Poitiers (number CER-TP 2019-11-08). The patients/participants provided their written informed consent to participate in this study.

## Author contributions

MA and NA coordinated the research project. TM, MA, NA, and AB elaborated the methodology of the study. TM, MA, and NA wrote the manuscript. TM and AB designed the cognitive and physical tasks. TM, AL, and FM collected the data. TM and MA analyzed the data. All authors contributed to the article and approved the submitted version.
