# Supplementary material for: A plausible link between the time-on-task effect and the sequential task effect
Source: Front Psychol. 2022 Oct 26;13:998393. doi: 10.3389/fpsyg.2022.998393 (PMC9643466; doi:10.3389/fpsyg.2022.998393)
Supplement: Supplementary file 1 [file Presentation_1.pdf]

Supplementary material

1. Determination of the sample size

Examining the correlation between the time-on-task effect and the sequential task effect requires to observe both effects in the same study and for the same participants. A previous experiment conducted with the same protocol (Mangin et al., 2021, exp. 3) required 51 participants to observe the sequential task effect and time-on-task effect. We tested the correlation on this sample of participants and obtained a marginal correlation: $r = .245$, $p = .081$. Then, a power analysis on G*Power was conducted to know how many participants it would be required to obtain a significant correlation with the following parameters: one tail, an alpha error probability of .05, and a power of .80. We obtained a sample size of 102 participants. Because of time and budget constraints we decided to conduct a study with 80 participants; i.e., a probability of .70 to obtain a significant correlation. Finally, we recruited 83 participants, but rejected 4 outlier participants.

2. Results concerning mean reaction time (RT) in the Stroop task while mixing the two types of trial

We conducted an ANOVA with Time-on-task (Part 1, Part 2, Part 3, Part 4) as repeated-measure factor on mean RT for correct responses in the Stroop task. We applied a correction of Greenhouse-Geisser on the degrees of freedom to compensate for any problem of sphericity. The effect of TOT was significant: F (2.2, 168.8) = 6.55; $p = .001$; $\eta^2_p = .077$. A post-hoc test with a correction of Holm showed that mean RT in Part 1 was marginally shorter than mean RT in Part 2 [t (234) = -2.39; $p = .071$; $d = 0.13$], and significantly shorter than mean RT in Part 3 [t (234) = -3.62; $p = .002$; $d = 0.19$] and Part 4 [t (234) = -4.02; $p < .001$; $d = 0.21$]. All other differences did not reach significance. Figure A1 illustrates this effect.

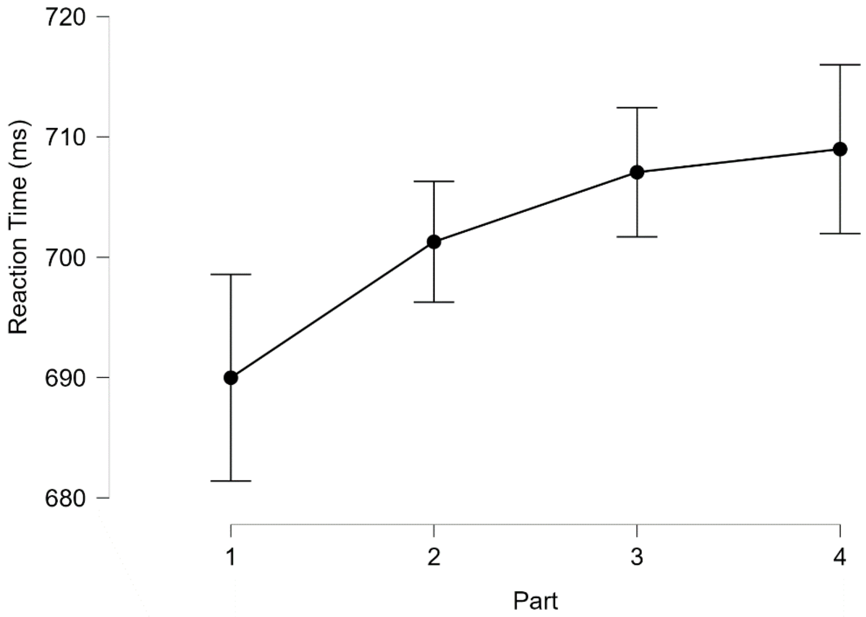

Figure A1: Effect of time-on-task in the Stroop task on mean RT for correct responses.

3. Results concerning error rates in the Stroop task while mixing the two types of trial

The same ANOVA design was applied to error rate in the Stroop task. The effect of TOT did not reach significance: *F* (2.24, 174.67) = 0.44; *p* = .668. The mean error rate was .068.

Results on mean RT and error rate showed that participants kept the same strategy during the whole task and that there was no speed-accuracy tradeoff over time.

4. Results concerning mean reaction time (RT) in the Stroop task while differentiating the two types of trial

An ANOVA with Time-on-task (Part 1, Part 2, Part 3, Part 4) and Type of trial (reading the color name, naming the ink color) as within-subjects factors was conducted on the reaction time during the Stroop task. The interaction between these two effects reached

significance: F (3, 234) = 19.29, $p < .001$, $\eta^2_p = .198$ (see Figure A2). A post-hoc test, revealed that for the 'naming ink color' trials, the part 1 (M = 757.65, SD = 89.56) was different from the three others (part 2, M = 779.86, SD = 90.65; part 3, M = 788.89, SD = 89.61; part 4, M = 794.02, SD = 87.66, all ps < .001). All the other comparisons concerning the 'naming ink color' trials did not reach significance (all *ps* > .072). For the 'reading color name' trials, none of the comparison reached significance (all *ps* > .600). These results support the fact that mental fatigue is closely related to cognitive control.

The main effect of Type of stimulus was significant: F (1, 78) = 570.02, $p < .001$, η^2_p = .880. Mean RT for 'reading color name' trials was consistently shorter than for 'naming ink color' trials regardless the part of the task (see Figure A2).

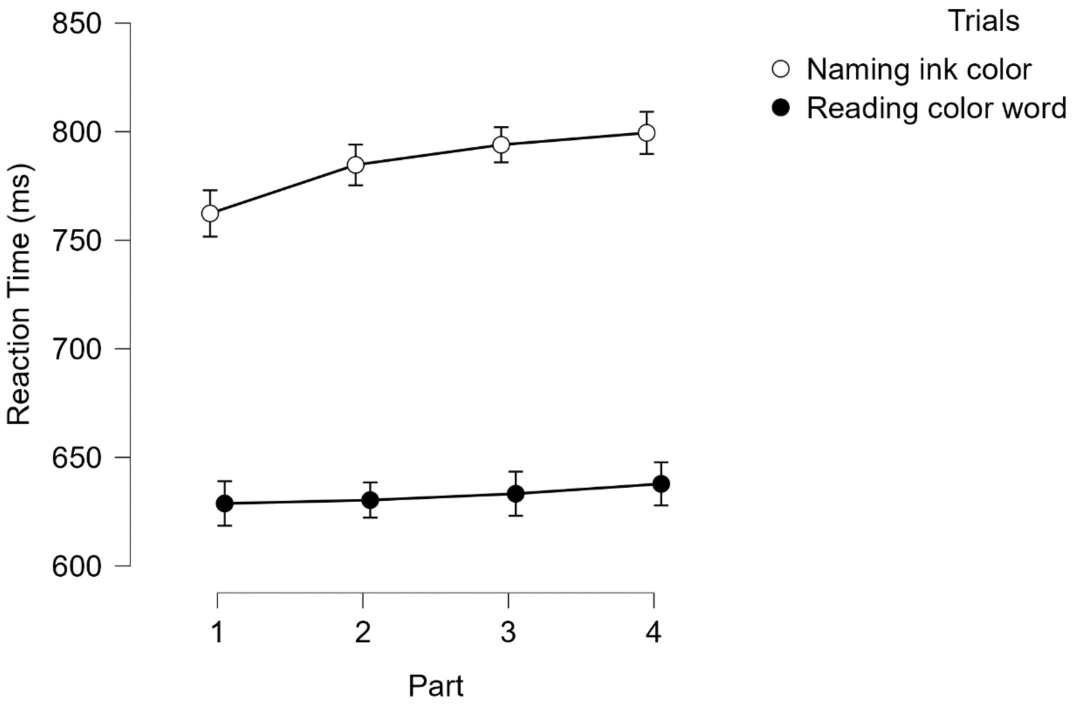

Figure A2: Mean reaction time in the incongruent Stroop task as a function of time-on-task and type of trials.

5. Results concerning error rates in the Stroop task while differentiating the two types of trial

An ANOVA with Time-on-task (Part 1, Part 2, Part 3, Part 4) and Type of trial (reading the color name, naming the ink color) as within-subjects factors was conducted on the accuracy during the Stroop task. The interaction and the main effect of TOT did not reach significance: F (3, 234) = 1.27, p = .285, η^2_p = .016, and F (3, 234) = 0.49, p = .689, η^2_p = .006, respectively. By contrast, the main effect of the Type of trial was significant: F (1, 78) = 68.98, p < .001, η^2_p = .469. Participants showed a higher accuracy for the ‘reading color name’ trials (M = 97.5%, SD = 2.8%) than for the ‘naming ink color’ trials (M = 91.1%, SD = 8.6%).

6. Correlations between subjective variables and behavioral phenomena of interest

A first series of correlations has been examined between subjective variables (boredom and fatigue) and behavioral effects of interest (time-on task effect, sequential task effect) while mixing the two types of trial for the time-on-task (TOT) effect. In this case the subjective score assessed just after the Stroop task was considered to calculate the correlation (and not the composite score as reported in the article). For these correlations the TOT effect was calculated by subtracting the IES scores of the fourth part from the first part of the Stroop task, and then divided this difference by the IES score of the first part.

TOT effect and boredom score after the Stroop task: r = -.348, p = .002.

Sequential task effect and boredom score after the Stroop task: r = -174, p = .126.

TOT effect and fatigue score after the Stroop task: r = -.184, p = .104.

Sequential task effect and fatigue score after the Stroop task: r = -.077, p = .503.

A second series of correlations has been examined between subjective variables (boredom and fatigue) and behavioral effects of interest (TOT effect, sequential task effect)

while differentiating the two types of trial for the time-on-task effect (see Table A1). For these correlations, the TOT effect was calculated by subtracting the mean reaction time for correct responses when the participants had to name the ink color of the fourth part from the first part of the Stroop task, and then divided this difference by the reaction time of the first part.

Table A1: Correlations between TOT effect and other variables of interest. * = significant correlation between the variables.

Variable	Time-on-task IES	Sequential task effect	Boredom	Fatigue
**Time-on-task RT**	r = .821<br>p < .001*	r = .270<br>p = .016*	r = -.312<br>p = .005*	r = -.132<br>p = .246

7. Mediation analysis

In order to be sure that boredom mediated the relationship between the time-on-task effect and the sequential task effect, we should first obtain a significant correlation between the three variables. As mentioned in Figure A2, the correlation between the boredom index (i.e., the difference between the boredom score after the Stroop task minus the boredom score after the Video task) and the sequential task effect was marginal, r = -.216, p = .055. By contrast, the correlation between the boredom index and the time-on-task effect was significant: r = -.332, p = .003. Then, a mediation analysis was conducted with bootstrap for 5000 samples. The total effect was significant, β (standardized) = 0.272, p = .013. The indirect effect was not significant: β = 0.047, p = .249. Finally, the direct effect, when controlling for the boredom index, was at the limit of significance: β = 0.25, p = .050. This made the mediation analysis inconclusive, because the direct effect was at the limit of significance.

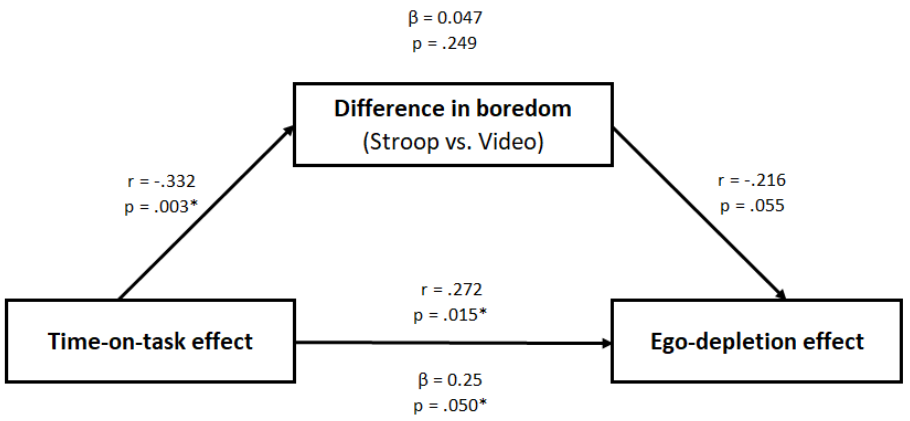

Figure A2: Mediation analysis between the time-on-task effect, the sequential task effect and the boredom index. * = significant correlation between two variables.

8. A systematic review of studies analyzing the time-on-task effect within the sequential task protocol

This systematic review aimed to examine if studies using the sequential task protocol with a long depleting task (duration ≥ 30 min) analyzed the performance in the depleting task as a function of time-on-task and the correlation between the time-on-task effect and the sequential task effect; two phenomena generally interpreted as manifestations of mental or cognitive fatigue.

To be included in the systematic review, a study should use the sequential task protocol with a depleting task and a control task longer than 20 min. The meta-analysis of Hagger et al. (2010) showed that the literature from social psychology mainly used short-duration depleting tasks (see appendix B of the Hagger's et al. meta-analysis). This meta-analysis included 198 experiments. A total of 25.3% of these experiments did not report the duration of the depleting task. The mean duration of the 140 remaining experiments was 6.27 min (SD = 3.22 min). Among these 140 experiments, only two used a depleting task with a duration of 20 and 30 min respectively (Tyler & Burns, 2009; Finkel et al., 2006 – exp. 5). None of these two

studies analyzed the performance of the depleting task as a function of time on task. Consequently, the systematic review focused on studies from sport sciences examining the effect of mental fatigue on a subsequent physical performance.

Sources that was used to identify studies for the systematic review included Academic Search Complete, CINAHL with full text, APA PsycINFO, and SPORTDiscuss with full text through the EBSCO platform. The search strategy was the following: AB ((mental N0 fatigue OR cognitive N0 fatigue) AND (control N0 condition OR control N0 treatment OR control N0 trial OR control N0 task)). The flow chart of the systematic review is represented on Figure A3. The selected studies are listed in Table A2. This table clearly showed that out of the 56 listed studies, 40 (71.43%) observed a sequential task effect on the performance of the dependent task, and only 10 (17.86%) reported a time-on-task effect on the performance of the depleting task. By contrast, only 5 studies (8.93%) observed a practice effect on the performance of the depleting task and 10 (17.86%) did not observe a significant effect on this dependent variable. Concerning our main hypothesis, only 8 studies (14.29%) observed both a time-on-task effect and a sequential task effect and were able to calculate a correlation between these two effects, but none of these 8 studies reported this correlation. The small sample size of these 8 studies ($10 < N < 25$; $M = 16.6$; $SD = 6.2$) can explain why they did not report the correlation between the two effects of interest. Finally, among the 56 studies listed in table A2, 32 (57.14%) did not report the performance of the depleting task as a function of time-on-task and consequently were not interested by the effect of mental fatigue that could be observed in the depleting task.

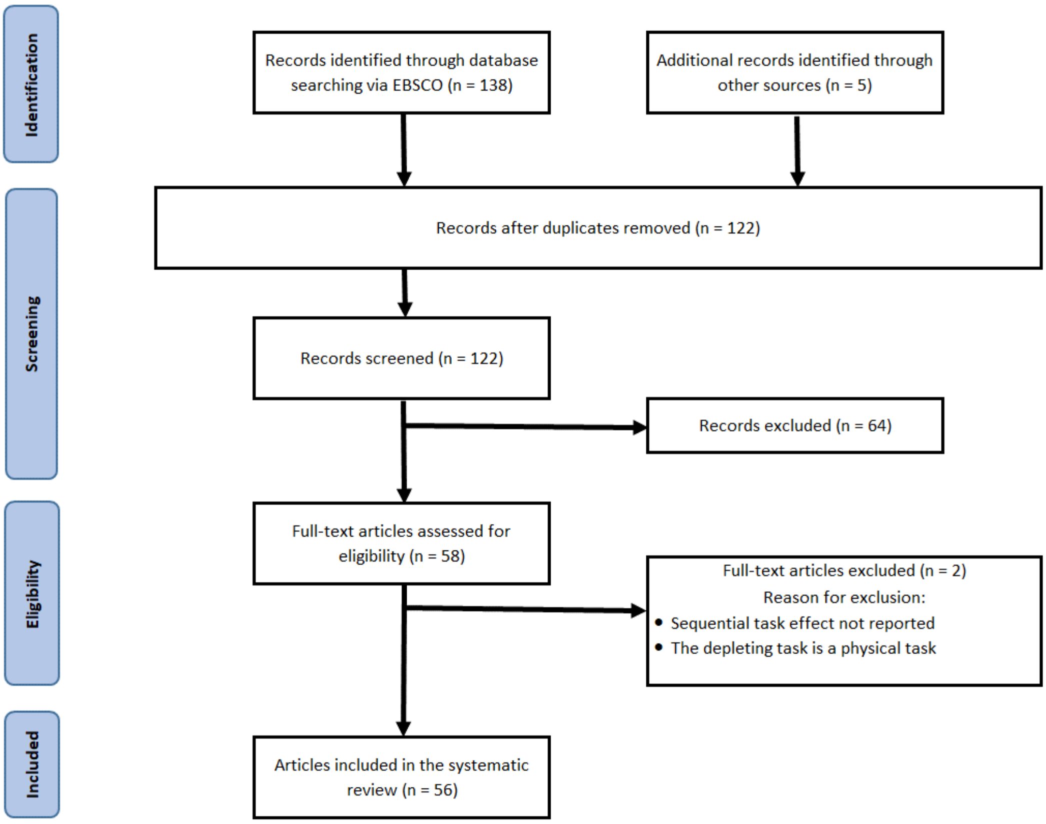

Figure A3: Flow chart of the systematic review on studies using the sequential task protocols and a depleting task with a duration above 20 minutes.

References

Azevedo, R., Silva-Cavalcante, M. D., Gualano, B., Lima-Silva, A. E. & Bertuzzi, R. (2016). Effects of caffeine ingestion on endurance performance in mentally fatigued individuals. *European Journal of Applied Physiology, 116*, 2293-2303. doi: 10.1007/s00421-016-3483-y

Badin, O. O., Smith, M. R., Conte, D., & Coutts, A. J. (2016). Mental fatigue: Impairment of technical performance in small-sided soccer games. *International Journal of Sports Physiology and Performance, 11*, 1100 -1105. http://dx.doi.org/10.1123/ijspp.2015-0710

Batista, M. M., Paludo, A. C., Da Silva, M. P., Martins, M. V., Pauli, P. H., Dal'maz, G., Stefanello, J. M., & Tartaruga, M. P. (2021). Effect of mental fatigue on performance, perceptual and physiological responses in orienteering athletes. *Journal of Sports Medicine and Physical Fitness, 61*(5), 673-679. doi: 10.23736/S0022-4707.21.11334-9

Brown, D. M. Y., & Bray, S. R. (2019). Effects of mental fatigue on exercise intentions and behavior. *Annals of Behavioral Medicine*, *53*, 405-414. doi: 10.1093/abm/kay052

Brownsberger, J., Edwards, A., Crowther, R., & Cottrell, D. (2013). Impact of mental fatigue on self-paced exercise. *International Journal of Sports Medicine*, *34*, 1029-1036. doi: 10.1055/s-0033-1343402

Budini, F., Labanca, L., Scholz, M., & Macaluso, A. (2022). Tremor, finger and hand dexterity and force steadiness, do not change after mental fatigue in healthy humans. *PLoS ONE, 17*(8), e0272033. https://doi.org/10.1371/journal.pone.0272033

Campos, B. T., Penna, E. M., Rodrigues, J. G. S., Mendes, T. T., Maia-Lima, A., Nakamura, F. Y., Vieira, E. L. M., Wanner, S. P., & Prado, L. S. (2021). Influence of mental fatigue on physical performance, and physiological and perceptual responses of judokas submitted to the special Judo Fitness Test. *Journal of Strength and Conditioning Research*, *36*(2), 461-468. doi: 10.1519/JSC.0000000000003453

Ciocca, G., Tessitore, A., Mandorino, M., & Tschan, H. (2022). Video-based tactical task does not elicit mental fatigue and does not impair soccer performance in a subsequent small-sided game. *Sports*, *10*, 31. https://doi.org/10.3390/sports10030031

Coutinho, D., Gonçalves, B., Wong, D. P., Travassos, B., Coutts, A. J., & Sampaio, J. (2018). Exploring the effects of mental and muscular fatigue in soccer players' performance. *Human Movement Science*, *58*, 287-296. https://doi.org/10.1016/j.humov.2018.03.004

Dallaway, N., Lucas, S. J. E., & Ring, C. (2022). Cognitive tasks elicit mental fatigue and impair subsequent physical task endurance: Effects of task duration and type. *Psychophysiology*, e14126, https://doi.org/10.1111/psyp.14126

Duncan, M. J., Fowler, N., George, O., Joyce, S. & Hankey, J. (2015). Mental fatigue negatively influences manual dexterity and anticipation timing but not repeated high-intensity exercise performance in trained adults. *Research in Sports Medicine*, *23*, 1-13. doi: 10.1080/15438627.2014.975811

Ferreira, A., Jr., Chierotti, P., Gabardo, J. M., Giovanini, B., Okano, A. H., Buzzachera, C. F., Okazaki, V. H. A., Okuno, N. M., & Altimari, L. R. (2020). Residual effects of mental fatigue on subjective fatigue, reaction time and cardiac responses. *Revista de Psicología del Deporte* [Journal of Sport Psychology], 29(2), 27-34.

Ferris, J. R., Tomlinson, M. A., Ward, T. N., Pepin, M. E., & Malek, M. H. (2021). Reduced electromyographic fatigue threshold after performing a cognitive fatiguing task. *Journal of Strength and Conditioning Research*, *35*(1), 267-274. DOI: 10.1519/JSC.0000000000002490

Filipas, L., Gallo, G., Pollastri, L., & La Torre, A. (2019). Mental fatigue impairs time trial performance in sub-elite under 23 cyclists. *PloS ONE, 14*(6), e0218405. https://doi.org/10.1371/journal.pone.0218405

Filipas, L., Borghi, S., La Torre, A., & Smith, M. R. (2021). Effects of mental fatigue on soccer-specific performance in young players. *Science and Medicine in Football*, *5*(2), 150-157. https://doi.org/10.1080/24733938.2020.1823012

Fortes, L. S., Berriel, G. P., Faro, H., Freitas-Júnior, C. G., & Peyré-Tartaruga, L. A. (2022a). Can prolongate use of social media immediately before training worsen high level male volleyball players' visuomotor skills? *Perceptual and Motor Skills*. https://doi.org/10.1177/00315125221123635

Fortes, L. S., Lima-Junior, D., Barbosa, B. T., Faro, H. K. C., Ferreira, M. E. C., & Almeida, S. S. (2022b). Effect of mental fatigue on decision-making skill and visual search behaviour in basketball players: An experimental and randomised study. *International Journal of Sport and Exercise Psychology*. doi: 10.1080/1612197X.2022.2058055

Gantois, P., Ferreira, M. E. C., de Lima-Junior, D., Nakamura, F. Y., Batista, G. R., Fonseca, F. S. & Sousa Fortes, L. de (2020) Effects of mental fatigue on passing decision-making performance in professional soccer athletes, *European Journal of Sport Science*, *20*(4), 534-543. https://doi.org/10.1080/17461391.2019.1656781

Gantois, P., Lima-Júnior, D. de, Fortes, L. de S., Batista, G. R., Nakamura, F. Y., & Fonseca, F. de S. (2021). Mental fatigue from smartphone use reduces volume-load in resistance training: A randomized, single-blinded cross-over study. *Perceptual and Motor Skills*, *128*(4), 1640-1659. https://doi.org/10.1177/00315125211016233

Gergelyfi, M., Sanz-Arigita, E. J., Solopchuk, O., Dricot, L., Jacob, B., & Zénon, A. (2021). Mental fatigue correlates with depression of task-related network and augmented DMN activity but spares the reward circuit. *NeuroImage*, *243*, 118532. https://doi.org/10.1016/j.neuroimage.2021.118532

Greco, G., Tambolini, R., Ambruosi, P., & Fischetti, F. (2017). Negative effects of smartphone use on physical and technical performance of young footballers. *Journal of Physical Education and Sport*, *17*(4), 2495-2501. doi:10.7752/jpes.2017.04280

Habay, J., Proost, M., De Wachter, J., Díaz-García, J., De Pauw, K., Meeusen, R., Van Cutsem, J., & Roelands, B. (2021). Mental fatigue-associated decrease in table tennis performance: Is there an electrophysiological signature? *International Journal of Environmental Research and Public Health*, *18*(24), 12906. doi: 10.3390/ijerph182412906

Hachard, B., Noé, F., Ceyte, H., Trajin, B., & Paillard, T. (2020). Balance control is impaired by mental fatigue due to the fulfilment of a continuous cognitive task or by the watching of a documentary. *Experimental Brain Research, 238*, 861-868. https://doi.org/10.1007/s00221-020-05758-2

Hakim, H., Khemiri, A., Chortane, O. G., Boukari, S., Chortane, S. G, Bianco, A., Marsigliante, S., Patti, A., & Muscella, A. (2022). Mental fatigue effects on the produced perception of effort and its impact on subsequent physical performances. *International Journal of Environmental Research and Public Health*, *19*(17), 10973. doi: 10.3390/ijerph191710973

Holgado, D., Troya, E., Perales, J. C., Vadillo, M. A., & Sanabria, D. (2021). Does mental fatigue impair physical performance? A replication study. *European Journal of Sport Science*, *21*(5), 762-770. doi: 10.1080/17461391.2020.1781265

Kosack, M. H., Staiano, W., Folino, R., Hansen, M. B., & Lønbro, S. (2020,). The acute effect of mental fatigue on badminton performance in elite players. *International Journal of Sports Physiology and Performance*, *15*, 632-638. https://doi.org/10.1123/ijspp.2019-0361

Kowalski, K. L., Tierney, B. C., & Christie A. D. (2022). Mental fatigue does not substantially alter neuromuscular function in young, healthy males and females. *Physiology & Behavior*, *253*, 113855. https://doi.org/10.1016/j.physbeh.2022.113855

Le Mansec, Y., Pageaux, B., Nordez, A., Dorel, S. & Jubeau, M. (2018). Mental fatigue alters the speed and the accuracy of the ball in table tennis. *Journal of Sports Sciences*, *36*(23), 2751-2759. https://doi.org/10.1080/02640414.2017.1418647

MacMahon, C., Schücker, L., Hagemann, N., & Strauss, B. (2014). Cognitive fatigue effects on physical performance during running. *Journal of Sport & Exercise Psychology*, *36,* 375-381. http://dx.doi.org/10.1123/jsep.2013-0249

Marcora, S. M., Staiano, W., & Manning, V. (2009). Mental fatigue impairs physical performance in humans. *Journal of Applied Physiology*, *106*, 857-864. doi: 10.1152/japplphysiol.91324.2008

Martin, K., Thompson, K. G., Keegan, R., Ball, N., & Rattray, B. (2015). Mental fatigue does not affect maximal anaerobic exercise performance. *European Journal of Applied Physiology*, *115*, 715-725. doi: 10.1007/s00421-014-3052-1

Martin, K., Staiano, W., Menaspà, P., Hennessey, T., Marcora, S., Keegan, R., Thompson, K. G., Martin, D., Halson, S., & Rattray, B. (2016). Superior inhibitory control and resistance to mental fatigue in professional road cyclists. *PLoS ONE, 11*(7), e0159907. doi: 10.1371/journal.pone.0159907

Moreira, A., Aoki, M. S., Franchini, E., da Silva Machado, D. G., Paludo, A. C., & Okano, A. H. (2018). Mental fatigue impairs technical performance and alters neuroendocrine and autonomic responses in elite young basketball players. *Physiology & Behavior, 196*, 112-118. https://doi.org/10.1016/j.physbeh.2018.08.015

Morris, A. J., & Christie, A. D. (2020). The effect of a mentally fatiguing task on postural balance control in young and older women. *Experimental Gerontology*, *132*, 110840. https://doi.org/10.1016/j.exger.2020.110840

Pageaux, B., Marcora, S. M., & Lepers, R. (2013). Prolonged mental exertion does not alter neuromuscular function of the knee extensors. *Medicine & Science in Sports & Exercise*, *45*(12), 2254-2264. doi: 10.1249/MSS.0b013e31829b504a

Pageaux, B., Lepers, R., Dietz, K. C., & Marcora, S. M. (2014). Response inhibition impairs subsequent self-paced endurance performance. European *Journal of Applied Physiology*, *114*, 1095-1105. doi: 10.1007/s00421-014-2838-5

Pageaux, B., Marcora, S. M., Rozand, V., & Lepers, R. (2015). Mental fatigue induced by prolonged self-regulation does not exacerbate central fatigue during subsequent whole-

body endurance exercise. *Frontiers in Human Neuroscience*, *9*, 67. doi: 10.3389/fnhum.2015.00067

Penna, E. M., Filho, E., Campos, B. T., Pires, D. A., Nakamura, F. Y., Mendes, T. T., Lopes, T. R., Smith, M. & Prado, L. S. (2018a) Mental fatigue does not affect heart rate recovery but impairs performance in handball players. *Revista Brasileira de Medicina do Esporte*, *24* (5), 347-351. http://dx.doi.org/10.1590/1517-869220182405180483

Penna, E. M., Filho, E., Wanner, S. P., Campos, B. T., Quinan, G. R., Mendes, T. T., Smith, M. R., & Prado, L. S. (2018b). Mental fatigue impairs physical performance in young swimmers. *Pediatric Exercise Science, 30*(2), 208-215. doi: 10.1123/pes.2017-0128

Persson, J., Larsson, A., & Reuter-Lorenz, P. A. (2013). Imaging fatigue of interference control reveals the neural basis of executive resource depletion. *Journal of Cognitive Neuroscience*, *25*(3), 338-351. https://doi.org/10.1162/jocn_a_00321

Pessoa, F. A., Pereira, L. C., de Oliveira Araújo, A., Oliveira, G. T. A., Pereira, D. C., & Elsangedy, H. M. (2022). Mental fatigue prior to aerobic exercise reduces exercise pleasure and negatively affects implicit attitudes toward future exercise. *Perceptual and Motor Skills*, *129*(3), 816-832. doi: 10.1177/00315125221091158

Queiros, V. S. de, Dantas, M., Fortes, L. de S., Silva, L. F. da, Silva, G. M. da, Dantas, P. M. S., & Cabral, B. G. de A. T. (2021). Mental fatigue reduces training volume in resistance exercise: A cross-over and randomized study. *Perceptual and Motor Skills*, *128*(1), 409-423. doi: 10.1177/0031512520958935

Rozand, V., Lebon, F., Papaxanthis, C., & Lepers, R. (2015). Effect of mental fatigue on speed-accuracy trade-off. *Neuroscience, 297,* 219-230. doi: 10.1016/j.neuroscience.2015.03.066

Salam, H., Marcora, S. M., & Hopker, J. G. (2018). The effect of mental fatigue on critical power during cycling exercise. European Journal of Applied Physiology, 118, 85-92. doi: 10.1007/s00421-017-3747-1

Schlichta, C., Cabral, L. L., da Silva, C. K., Bigliassi, M., & Pereira, G. (2022). Exploring the impact of mental fatigue and emotional suppression on the performance of high-intensity endurance exercise. *Perceptual and Motor Skills*, *129*(4), 1053-1073. doi: 10.1177/00315125221093898

Slimani, M., Znazen, H., Bragazzi, N. L., Zguira, M. S., & Tod, D. (2018). The effect of mental fatigue on cognitive and aerobic performance in adolescent active endurance athletes: Insights from a randomized counterbalanced, cross-over trial. *Journal of Clinical Medicine*, *7*(12), 510. doi: 10.3390/jcm7120510

Smith, M. R., Marcora, S. M., & Coutts, A. J. (2015). Mental fatigue impairs intermittent running performance. *Medicine & Science in Sports & Exercise*, *47*(8), 1682-1690. doi: 10.1249/MSS.0000000000000592

Smith, M. R., Coutts, A. J., Merlini, M., Deprez, D., Lenoir, M., & Marcora, S. M. (2016). Mental fatigue impairs soccer-specific physical and technical performance. *Medicine & Science in Sports & Exercise*, *48*(2), 267-276. doi: 10.1249/MSS.0000000000000762

Staiano, W., Bosio, A., Piazza, G., Romagnoli, M., & Invernizzi, P. L. (2019). Kayaking performance is altered in mentally fatigued young elite athletes. *The Journal of Sports Medicine and Physical Fitness*, 7. doi: 10.23736/S0022-4707.18.09051-5

Trecroci, A., Boccolini, G., Duca, M., Formenti, D., & Alberti, G. (2020). Mental fatigue impairs physical activity, technical and decision-making performance during small-sided games. *PLoS ONE, 15*(9), e0238461. https://doi.org/10.1371/journal.pone.0238461

Van As, S., Beckers, D. G. J., Geurts, S. A. E., Kompier, M. A. J., Husain, M., & Veling, H. (2021). The impact of cognitive and physical effort exertion on physical effort decisions: A pilot experiment. *Frontiers in Psychology, 12*, 645037. doi: 10.3389/fpsyg.2021.645037

Van Cutsem, J., De Pauw, K., Buyse, L., Marcora, S., Meeusen, R., & Roelands, B. (2017). Effects of mental fatigue on endurance performance in the heat. *Medicine & Science in Sports & Exercise, 49*(8), 1677-1687. doi: 10.1249/MSS.0000000000001263

Van Cutsem, J., De Pauw, K., Vandervaeren, C., Marcora, S., Meeusen, R., & Roelands, B. (2019). Mental fatigue impairs visuomotor response time in badminton players and controls. *Psychology of Sport & Exercise, 45,* 101579. https://doi.org/10.1016/j.psychsport.2019.101579

Van Cutsem, J., Van Schuerbeek, P., Pattyn, N., Raeymaekers, H., De Mey, J., Meeusen, R., & Roelands, B. (2022). A drop in cognitive performance, whodunit? Subjective mental fatigue, brain deactivation or increased parasympathetic activity? It's complicated! *Cortex, 155,* 30-45. doi: 10.1016/j.cortex.2022.06.006

Veness, D., Patterson, S. D., Jeffries, O., & Waldron, M. (2017). The effects of mental fatigue on cricket-relevant performance among elite players. *Journal of Sports Sciences,* 35(24), 2461-2467. https://doi.org/10.1080/02640414.2016.1273540

Verschueren, J. O., Tassignon, B., Proost, M., Teugels, A., Van Cutsem, J., Roelands, B., Verhagen, E., & Meeusen, R. (2020). Does mental fatigue negatively affect outcomes of functional performance tests? *Medicine & Science in Sports & Exercise, 52*(9), 2002-2010. doi: 10.1249/MSS.0000000000002323

Weerakkody, N. S., Taylor, C. J., Bulmer, C. L., Hamilton, D. B., Gloury, J., O'Brien, N. J., Saunders, J. H., Harvey, S., & Patterson, T. A. (2021). The effect of mental fatigue on the performance of Australian football specific skills amongst amateur athletes. *Journal*

of Science and Medicine in Sport, *24*, 592-596.

https://doi.org/10.1016/j.jsams.2020.12.003

Table A2: List of studies using the sequential task protocols included in the systematic review.

#	Authors	N	Depleting task	Task duration	TOT effect	Control task	Dependent task	ST effect	Correlation between TOT and ST effects
1	Marcora et al. (2009)	16	AX-CPT	90 min	Yes	Watching a documentary	Time to exhaustion while cycling at 80% PPO	Yes	Not reported
2	Brownsberger et al. (2013)	12	Vigilance task consisting responding to selected letters	90 min	NR	Watching a documentary	Two bouts of self-paced cycling exercise at RPE 11 and RPE 15	Yes	Not reported
3	Pageaux et al. (2013)	10	AX-CPT	90 min	No	Watching a documentary	Time to exhaustion while performing an isometric contraction at 20% MVC	Yes	Not reported
4	Persson et al. (2013)	32	High interference recognition task	> 20 min	NR	Low interference recognition task	Verb Generation Task	Yes	Not reported
5	MacMahon et al. (2014)	20	AX-CPT	90 min	No	Watching a documentary	Time to perform a 3-km run	Yes	Not reported
6	Pageaux et al. (2014)	12	Incongruent Stroop task	30 min	No	Congruent Stroop task	Time to perform a 5-km run	Yes	Not reported
7	Duncan et al. (2015)	8	Vigilance task consisting completing concentration grids	40 min	NR	Watching a documentary	Four 30-second Wingate Anaerobic Tests + Coincidence anticipation timing task + Minnesota Manual Dexterity Turning Test	Yes	Not reported
8	Martin et al. (2015)	12	AX-CPT	90 min	No	Watching a documentary	Countermovement jump, isometric leg extension,	No	Not reported

							and 3 min all out cycle test		
9	Pageaux et al. (2015)	12	Incongruent Stroop task	30 min	No	Congruent Stroop task	6-min bout of cycling at 80% PPO	No	Not reported
10	Rozand et al. (2015)	10	Incongruent Stroop task	90 min	NR	Watching a documentary	Movement time in a Fitt's pointing task	Yes	Not reported
11	Smith et al. (2015)	10	AX-CPT	90 min	Yes	Watching a documentary	Running velocity in an intermittent running exercise	Yes	Not reported
12	Azevedo et al. (2016)	8	AX-CPT	90 min	NR	Watching a documentary	Endurance capacity in a cycling exercise at 80 % MPO	No	Not reported
13	Badin et al. (2016)	20	Stroop task	30 min	NR	Watching a documentary	Technical performance in small-sided soccer games	Yes	Not reported
14	Martin et al. (2016)	11 PC + 9 RC	Incongruent Stroop task	30 min	PE	10-min at focusing on a centered black cross	Power output in a 20-min time trial on a cycle ergometer	Yes, in RC only	Not reported
15	Smith et al. (2016) Study 2	14	Incongruent Stroop task	30 min	NR	Reading magazines	Loughborough soccer passing and shooting tests	Yes	Not reported
16	Greco et al. (2017)	16	Use of smartphones (Brain It on App)	30 min	NR	Carrying out usual activities before training	Yo-Yo Intermittent Recovery Test + Loughborough Soccer Passing Test	Yes	Not reported
17	Van Cutsem et al. (2017)	10	Incongruent Stroop task	45 min	No	Watching a documentary	Flanker task + 45-min bout of cycling at 60% + 15-min bout of cycling at 80%	No	Not reported

18	Veness et al. (2017)	10	Incongruent Stroop task	30 min	Yes	Reading magazines	Cricket run-two test + Batak Lite hand-eye coordination test + Yo-Yo-IR1 test	Yes	Not reported
19	Coutinho et al. (2018)	10	Incongruent Stroop task	30 min	NR	No specific treatment before	Small-sided games	Yes	Not reported
20	Le Mansec et al. (2018)	22	AX-CPT	90 min	NR	Watching a movie	Table tennis performance	Yes	Not reported
21	Moreira et al. (2018)	32	Incongruent Stroop task	30 min	PE	10-min at focusing on a centered black cross	Small-Sided-Games (SSG) technical performance	Yes	Not reported
22	Penna et al. (2018a)	12	Modified Stroop task	30 min	NR	Watching a documentary	Yo-Yo IR1 test	Yes	Not reported
23	Penna et al. (2018b)	16	Incongruent Stroop task	30 min	NR	Watching a documentary	1500-m time trial of swimming	Yes	Not reported
24	Salam et al. (2018)	11	Incongruent Stroop task	30 min	NR	Reading a magazine	Time to exhaustion while cycling at 40%, 60%, 80% or 100% $\dot{V}O_{2peak}$	Yes	Not reported
25	Slimani et al. (2018)	10	Incongruent Stroop task	30 min	NR	Reading magazines	d2 test + 20-m multistage fitness test	Yes	Not reported
26	Brown et al. (2019)	25	AX-CPT	50 min	Yes	Watching a documentary	30-min cycling exercise at a self-selected intensity	Yes	Not reported
27	Staiano et al. (2019)	13	Incongruent Stroop task	60 min	NR	Watching a documentary	2000-m kayaking time trial	Yes	Not reported
28	Filipas et al. (2019)	23	Incongruent Stroop task	30 min	No	Watching a documentary	Distance covered in a 30-min cycling exercise	Yes	Not reported
29	Van Cutsem et al. (2019)	11 HC + 9 BP	Incongruent Stroop task	90 min	Yes	Watching a documentary	Flanker task + Visuomotor task	Yes	Not reported

30	Ferreira et al. (2020)	20	Modified incongruent Stroop task	30 min	NR	Seating while receiving no stimuli	Reaction time task	Yes	Not reported
31	Gantois et al. (2020)	20	Stroop task	30 min	NR	Watching advertising videos	Passing decision-making task + Stroop task	Yes	Not reported
32	Hachard et al. (2020)	20	AX-CPT	90 min	Yes	Watching a documentary	Postural tasks	Yes	Not reported
33	Kosack et al. (2020)	19	Incongruent Stroop task	60 min	PE	Watching a documentary	Badminton-Specific Test + Countermovement-jump	No	Not reported
34	Morris & Christie (2020)	16 YA + 16 OA	Psychomotor vigilance task	20 min	Yes in YA	Watching a documentary	Postural task	Yes in YA	Not reported
35	Verschueren et al. (2020)	14	Incongruent Stroop task	90 min	No	Watching a documentary	Neurocognitive functional performance tests	Yes	Not reported
36	Batista et al. (2021)	15	Stroop task	30 min	NR	10-min at focusing on a centered black cross + 20 min at relaxing	Time to perform an orienteering race	No	Not reported
37	De Queiros et al. (2021)	10	Stroop task	30 min	NR	Watching a documentary	Countermovement jump + Half-back squat exercise	Yes	Not reported
38	Ferris et al. (2021)	8	AX-CPT	60 min	NR	Watching a documentary	Incremental single-leg knee-extensor ergometry test	Yes	Not reported
39	Filipas et al. (2021)	12 U14 + 12 U16 + 12 U18	Incongruent Stroop task	30 min	No	Reading magazines	Loughborough Soccer Passing and Shooting Tests	Yes	Not reported

40	Gantois et al. (2021)	20	Social media activity through smartphone apps	30 min	NR	Watching a documentary	Three sets of Back-squat test with 80% of 15RM	No	Not reported
41	Gergelyfi et al. (2021)	26	Modified Stroop task	90 min	NR	Reading health magazines	Missing Number task + Simple reaction time task	Yes	Not reported
42	Habay et al. (2021)	11	Incongruent Stroop task	60 min	No	Watching a documentary	Flanker task + Visuomotor task	Yes	Not reported
43	Holgado et al. (2021)	30	AX-CPT	90 min	NR	Watching a documentary	Time to exhaustion while cycling at 80% VO_{2max}	No	Not reported
44	Van As et al. (2021)	20	2-back task	45 min	NR	Watching a documentary	Effort-Based Decision-Making task	No	Not reported
45	Weerakkody et al. (2021)	25	Stroop task	30 min	Yes	Watching a documentary	Standing vertical jump + running vertical jump + AFL agility test + 20 m-sprint test + Matthew Lloyd clean hands test + Brad Johnson goal kicking test + Yo-Yo IR1	Yes	Not reported
46	Budini et al. (2022)	29	Switch task	100 min	NR	Watching a movie	Postural, isometric and kinetic tremor, pinch force and steadiness tests + Purdue pegboard test	No	Not reported
47	Campos et al. (2022)	13	Incongruent Stroop task	30 min	NR	Watching a documentary	Special Judo Fitness Test	No	Not reported
48	Ciocca et al. (2022)	10	Video-based tactical task	30 min	NR	Watching a documentary	Small-sided game	No	Not reported
49	Dallaway et al. (2022)	90	Incongruent Stroop task / 2-back task	40 min	PE	Watching a documentary	Self-paced rhythmic handgrip exercise	Yes	Not reported

50	Fortes et al. (2022a)	18	Using social media on a smartphone	30 min	NR	Watching a documentary	Visuomotor Task	Yes	Not reported
51	Fortes et al. (2022b)	16	Sport-based video games	60 min	NR	Watching a documentary	Incongruent Stroop task + Decision-making task	Yes	Not reported
52	Hakim et al. (2022)	12	Stroop task	60 min	NR	Watching a documentary	20-min bout of cycling at a level of 13 on the Borg's scale	No	Not reported
53	Kowalski et al. (2022)	30	Psychomotor Vigilance Task	30 min	Yes	Watching a documentary	10 s isometric dorsiflexion contractions at 10, 20 and 50% MVC	No	Not reported
54	Schlichta et al. (2022)	12	Incongruent Stroop task<br>Emotion Suppression task	30 min	NR	Seating in a comfortable chair	Cycling at 80% PPO until exhaustion	Yes	Not reported
55	Trecroci et al. (2022)	9	Stroop task	30 min	NR	Watching a documentary	Small-sided games	Yes	Not reported
56	Van Cutsem et al. (2022)	20	Incongruent Stroop task	90 min	Yes, for CS<br>PE for MS	Watching a documentary	Flanker task	No	Not reported

Note: BP = Badminton players; CS = Color stimuli; HC = Healthy controls; MPO = Maximal power output; MS = Meaning stimuli; MVC = Maximal voluntary contraction; NR = Not reported; OA = Older adults; PC = Professional cyclists; PPO = Peak power output; RC = Recreational cyclists; ST = Sequential task; PE = Practice effect; TOT = Time-on-task; U14 = Soccer players under 14 years; U16 = Soccer players under 16 years; U18 = Soccer players under 18 years; YA = Young adults.
